# How word meaning structure relates to executive functioning and theory of mind in children with developmental language disorder: A multiple case study

**DOI:** 10.1177/23969415241268245

**Published:** 2024-08-21

**Authors:** Thomas F. Camminga, Daan Hermans, Eliane Segers, Constance T.W.M. Vissers

**Affiliations:** 404818 Radboud University, Behavioural Science Institute, Nijmegen, the Netherlands; 404818 Radboud University, Behavioural Science Institute, Nijmegen, the Netherlands; 100511 Royal Kentalis, Sint-Michielsgestel, the Netherlands; 404818 Radboud University, Behavioural Science Institute, Nijmegen, the Netherlands; 404818 Radboud University, Behavioural Science Institute, Nijmegen, the Netherlands; 100511 Royal Kentalis, Sint-Michielsgestel, the Netherlands

**Keywords:** Developmental language disorder, word meaning structure, executive functioning, theory of mind, behavioral problems

## Abstract

**Background and aims:**

Many children with developmental language disorder (DLD) have difficulties in executive functioning (EF) and theory of mind (ToM). These difficulties might be explained by the theory that children's conceptual understanding changes over five stages of word meaning structure, from concrete and context-dependent to abstract and precise. We present a multiple case study examining how word meaning structure relates to EF and ToM in children with DLD.

**Methods:**

Participants were five children with DLD aged 9–12 and five typically developing children matched for age, gender, and nonverbal intelligence. Word meaning structure was assessed using new dynamic test. EF was assessed using the Zoo Map Task and Behavioral Rating Inventory for EF. ToM was assessed using the ToM test, Frith-Happé Animations, and Bermond-Vorst Alexithymia Questionnaire. Behavioral problems were measured using the Child Behavior Checklist. Anamnestic interviews with the parents were conducted to describe the case histories.

**Results:**

For the children with DLD, lower scores in the word meaning structure task were observed compared to those observed for their matched peers, with no statistical test applied. Word meaning structure related positively to EF and ToM, but not to behavioral problems. Instances in which word meaning structure dissociates from EF and ToM are discussed in individual case descriptions.

**Conclusions:**

By linking language to conceptual development, variations in word meaning structure may explain some EF and ToM difficulties in children with DLD.

**Implications:**

The present study offers a basis for future research on the relationships among word meaning structure, EF, and ToM.

## Introduction

Developmental language disorder (DLD) is characterized by difficulties in developing age-appropriate expressive and receptive language skills that are not associated with known biomedical causes (Bishop et al., 2017). Speaking difficulties may be seen in delayed language milestones, limited speech output, and use of basic sentences. Comprehension issues may include inattentiveness, difficulty following instructions, misunderstanding, or rapid forgetfulness of information (Norbury et al., 2024). These verbal difficulties may extend to the internal functions of language in cognition (Alderson-Day & Fernyhough, 2015; Toomela, 2020a), including in executive functioning (EF) and theory of mind (ToM).

In addition to language problems, many children with DLD have difficulties with EF (Pauls & Archibald, 2016) and ToM (Nilsson & de López, 2016). EF and ToM have been connected to behavioral problems (e.g., Carter Leno et al., 2021; Schoemaker et al., 2013). Indeed, according to a meta-analysis by Hentges et al. (2021), both externalizing (e.g., aggression or rule breaking) and internalizing (e.g., social withdrawal) problems are relatively prevalent in children with DLD. Although these problems are often ascribed predominantly to social-communicative difficulties (e.g., Forrest et al., 2021), EF and ToM difficulties in children with DLD may also in part explained by the theory that language has an internal role in cognition (Alderson-Day & Fernyhough, 2015; Vygotsky, 1934). Words contribute to conceptual development because words acquire their meaning due to their connection to mental representations (e.g., a mental “image” of a dog; Toomela, 2020a). As children age, their conceptual understanding changes over the course of five stages of word meaning structure, whereby the meanings of words develop from concrete and context-dependent (e.g., “sadness is when I cry”) to increasingly abstract and precise (e.g., “sadness is a negative emotion”; Toomela, 2020b). Since EF and ToM rely on conceptual understanding, both may benefit from word meaning structure development (Camminga et al., 2021). We conducted a multiple case study that examines the word meaning structure of children with DLD in order to assess whether their EF and ToM difficulties may indeed be related to the internal role of language in cognition.

### EF and ToM in children with DLD

#### EF

EF consists of higher cognitive processes used to actively engage, direct, or coordinate various lower cognitive processes, usually in the pursuit of specific goals (Doebel, 2020). EF comes into play when individuals exert control over their thoughts and actions, particularly in situations where they are attempting tasks that clash with ingrained habits, impulses, or desires. For example, in school, children often need to sustain their attention on a given task that is not immediately rewarding in the face of various sources of distraction. EF development starts with elementary functions such as inhibitory control and working memory (Diamond, 2013). Hierarchically, building upon these elementary functions, more complex components of EF emerge, such as flexible task switching, problem-solving, and planning. Relying on the involvement of lower components of EF, planning abilities allow children to systematically organize their actions in distinct steps that need to be taken in order to attain a goal in the distant future. EF can protect against behavioral problems (for a meta-analysis of longitudinal studies, see Yang et al., 2022) by inhibiting impulsive or inappropriate actions, shifting attention away from negative stimuli, and modifying goals and plans based on the needs, goals, impulses, and emotions of others (Vissers & Hermans, 2018).

Children with DLD experience difficulties with EF, which may be partly explained by the relationship between language and EF. EF difficulties are already evident in preschool children with DLD (for a review see Vissers et al., 2015), who already score lower on performance measures of inhibitory control (e.g., Thomas et al., 2022), working memory (e.g., Marton, 2008), and cognitive flexibility (e.g., Blom et al., 2021). Meta-analyses of school-age children have revealed that TD children outperform children with DLD in terms of inhibitory control, cognitive flexibility, and visuospatial working memory (Pauls & Archibald, 2016; Vugs et al., 2013). Children with DLD also appear to have difficulties with hierarchically more complex EF processes such as planning (Aziz et al., 2017; Lidstone et al., 2012). The role of language in EF is suggested by studies based on cross-lagged analysis in TD children show that early language development generally (Romeo et al., 2022; Slot & von Suchodoletz, 2018), and expressive vocabulary specifically (Jones et al., 2020) predict later EF ability to a greater extent than early language development predicts later EF. Moreover, deaf children who often have impaired language abilities due to sensory rather than cognitive factors than TD children, have EF difficulties that are mediated by their language abilities (Botting et al., 2017; Merchán et al., 2022); the reverse effect of mediation (from EF to language) was not found (Botting et al., 2017).

#### ToM

ToM is defined as the ability to understand the behavior of others in mental terms (Kanske, 2018; Premack & Woodruff, 1978). Over the course of development children learn that others peoples thoughts, goals, beliefs, desires, and emotions may differ from their own. At the start of ToM development, children learn imitation skills, joint attention, and emotion recognition (Westby & Robinson, 2014). From the age of four, first-order ToM emerges, meaning that from this age, children learn to reason about others’ mental states. This is first indicated by the finding that, from this age, children begin to understand that others can have false beliefs (Wellman et al., 2001). From around the age of 6, some children can consider what one person is thinking about the mental state of another person. ToM continues to develop beyond the age of seven (see review in Lagattuta et al., 2015). As they reach the age of 7, children begin developing the ability to reflect on and intentionally influence their own and others’ thought processes. ToM plays a role in preventing behavioral problems (Holl et al., 2021) by supporting the proper interpretation of social situations, thereby preventing interpersonal aggression (Choe et al., 2013).

Children with DLD have difficulties with ToM, which—as with EF—appear to draw upon language to a certain degree. Preschoolers with DLD already score lower on various early facets of ToM development (for a review, see Vissers & Koolen, 2016), including joint attention, imitation (Farrant et al., 2011), emotion recognition (e.g., Löytömäki et al., 2020; Taylor et al., 2015), and understanding (Spackman et al., 2006). Strong evidence for first-order ToM difficulties in children with DLD comes from a meta-analysis that revealed that children with DLD in primary school are outperformed by TD children on false-belief tasks by almost one standard deviation (Nilsson & de López, 2016). Another meta-analysis revealed an association between language difficulties and alexithymia, a subclinical condition characterized by poor emotional awareness (Lee et al., 2022). There are several indications that theory that language plays an internal role in ToM. Language ability generally, and particularly syntax specifically, are both strongly associated with ToM in children below the age of 7 (Milligan et al., 2007), and language mediates the relation between age and ToM (Bigelow et al., 2021). Moreover, deaf children with hearing parents have ToM difficulties, whereas this is not the case to the same extent for deaf children with deaf parents (Schick et al., 2007) and children with early hearing provisions (Yu et al., 2021).

### Linking language to conceptual development: Word meaning structure and its relation to EF and ToM

To date, no theory has considered the full scope of the EF and ToM difficulties in children with DLD. Distinct aspects of language development have been proposed to contribute in specific ways to aspects of EF and ToM (de Villiers & de Villiers, 2014). For example, complementation syntax (e.g., “Sammy thinks that *Santa is in town*”; complement in italics) is thought to allow for distinguishing beliefs from reality. However, these theories leave open the question of why children with DLD have wide-ranging difficulties in EF and ToM. For example, although complementation syntax can partially account for difficulties in false-belief understanding, it cannot explain why children with DLD have difficulties in emotion recognition (e.g., Löytömäki et al., 2020). Since EF and ToM rely on conceptual understanding (Doebel, 2020; Wellman, 2018), difficulties in EF and ToM in children with DLD may emerge from a connection between language and conceptual development that is not grasped within traditional measures of language, such as vocabulary or syntactic tests (Toomela, 2020b).

The relationship between language and conceptual development can be found in the concept of word meaning structure (Toomela, 2020b). A structure is a whole composed of elements in specific relationships; word meaning structure refers to the system of interrelated elements of word meaning. The meaning of a word is constituted by a mental representation (e.g., a mental “image” of a dog) or constructed from sensory attributes grounded in experiences. This representation is connected to another sensory pattern, such as a word or a gesture (e.g., the word “dog”), which stands in a referential relation to the representation. Over the course of development, words increasingly differentiate from each other into different classes, and from the representations they refer to.^1^

### Stages of word meaning structure

Following Vygotsky's (1934) early work, Toomela (2020b) distinguished five stages of word meaning structure. A critical development in word meaning structure with respect to EF and ToM occurs during the transition between two of these stages (Table 1). From the age of 3, children gradually develop *everyday concepts*. Words at this stage refer directly to sensory attributes. The category boundaries of everyday concepts are fuzzy and based on perceptual similarity and everyday situations. By contrast, *logical concepts*, which begin to emerge from the age of 7, are structured hierarchically. At this stage, linguistic symbols can refer to other symbols, instead of directly linking them to sensory attributes. This facilitates categorization on a nonsensory basis, enabling the grouping, analysis, and verbal expression of items that differ in their appearances and contextual associations. For example, a child may change its understanding of a tree from “a brown thing that you can climb on” (everyday concept), to “a living being” (logical concept). Although word meaning structure develops in stages, individuals may at one point in development rely on everyday concepts for one area (e.g., for interpersonal concepts), but on logical concepts for another area (e.g., for scientific concepts). For example, a single individual may rely on everyday concepts in the area of interpersonal relations, but think based on logical concepts in scientific areas such as physics (Toomela, 2020b). Therefore, the trajectory from one stage to the next can be viewed as a continuum.

**Table 1.** Differences Between Everyday Concepts and Logical Concepts.

	Everyday concepts	Logical concepts
Age of first emergence	May first emerge from 3 years old.	May first emerge from 7 years old.
Referents	Word refer directly to sensory attributes.	Words may refer to other words, and thus only indirectly to sensory attributes.
Category boundaries	Categories have fuzzy boundaries based on perceptual similarities and everyday situations.	Categories have strict, verbally defined boundaries.
Supported thought processes	Thinking is bounded by concrete experiences.	Abstract problem solving becomes possible, in a purely verbal context.
Example	“Whales are big and swim in the sea.”	“A whale is a mammal because female whales have mammary glands.”
How they support executive functioning	Representing verbal behavioral rules and multistep plans that stretch unto the far future.	Efficient self-regulation and abstract planning.
How they support theory of mind	Understanding of mental states in concrete terms (e.g., “knowing” means having seen with your own eyes).	Accurate and abstract understanding of the processes underlying mental states.

Word meaning structure has, so far, not been studied directly in children with DLD. Nevertheless, there are some indirect indications that children with DLD diverge in their word meaning structure development compared to TD children (Acosta Rodríguez et al., 2020; Dosi, 2021; Dosi & Gavriilidou, 2021). Children with DLD find it more difficult to comprehend categorical relationships between words (e.g., Acosta Rodríguez et al., 2020). In the domain of definitional skills, several studies have shown that children with DLD tend to offer more concrete and informal word definitions compared with TD children (Dosi & Gavriilidou, 2021; Marinellie & Johnson, 2002). Moreover, Dosi (2021) showed that definitional skills correlated with aspects of EF, namely working memory, updating, and inhibition, in TD children but not in children with DLD.

### Relation of word meaning structure to EF and ToM

Theoretically, the trajectory from everyday concepts to logical concepts may affect EF and ToM (Camminga et al., 2021; Toomela, 2020b). In the domain of EF, language allows individuals to verbalize and organize their behavioral plans (Zelazo, 2020). Logical concepts, which permit reflection on and manipulation of words independent of sensory experiences, may facilitate the construction of more abstract and flexible plans. Moreover, owing to their sharp boundaries, logical concepts allow children to regulate their cognitive processes with increased precision (Toomela et al., 2020b). In the domain of ToM, logical concepts may allow children to consciously analyze verbally expressed thought processes from themselves and others. Moreover, the sharp boundaries of logical concepts may allow for a more precise taxonomy of mental concepts. For example, in the emotion recognition domain, fine-grained emotion concepts increase the precision of emotion recognition (Gendron et al., 2012).

### Measuring word meaning structure

Recently, researchers have measured word meaning structure using tasks that assess the ability of individuals to define and hierarchically classify concepts under a higher order term (e.g., Murnikov & Kask, 2021; Toomela et al., 2020a). For example, a participant may be asked, “Why do a cat and a dog go together”? These tasks are usually presented verbally, without the assistance of the experimenter. However, such a static approach can only assess which stage of word meaning structure is dominant at a specific moment in a specific conceptual domain (Hasson & Joffe, 2007). It is possible though that a single child has developed logical concepts but that they are overshadowed by more dominant everyday conceptual responses. In line with dynamic assessment approaches (Hasson & Joffe, 2007), Luria (1976) gradually increased support if participants were unable to classify concepts hierarchically. Thus, this method can be useful for distinguishing between (a) logical concepts that are activated instantly (i.e., dominant logical concepts), (b) logical concepts that have been developed but are overshadowed by everyday conceptual responses (i.e., latent logical concepts), and (c) the absence of logical concepts (i.e., everyday concepts).

### The present study

Based on an overview of the literature, there are theoretical arguments for assuming that EF and ToM difficulties in children with DLD can be accounted for by word meaning structure. No studies have compared word meaning structure between children with DLD and TD children. Similarly, the contributions of word meaning structure to EF and ToM, and by extension behavioral problems of these children have not been investigated. This study aimed to provide a first step towards answering the following research questions:

1. How do children with DLD differ from TD children in their word meaning structure?
2. What is the association between word meaning structure in children with DLD and TD children with (a) EF, (b) ToM, and (c) behavioral problems?

We considered a small-scale approach that mainly focused on the individual level as a prudent first step for two reasons. First, there has been limited exploration of these themes in prior research. Second, due to traditional “diagnosis-by-exclusion,” the DLD population is highly heterogeneous (Tomas & Vissers, 2019). This implies that findings that apply to the group do not always apply at the individual level. A multiple case study allows us to highlight individual differences. We describe five case studies involving children between the ages of 9 and 12. This age range represents a point where logical concepts develop rapidly and individual differences can emerge (Kask et al., 2019). We show how individual children fit into the general pattern of relationships between word meaning structure, EF, ToM, and behavioral problems.

## Method

### Participants

This study was approved by the Ethics Committee of the Faculty of Social Sciences of the Radboud University (21N.004982). The participants were five children with DLD (age: *M *= 10.9, *SD *= 1.1) and five TD children (age: *M *= 10.8, *SD *= 1.2). Three of the children with DLD were recruited via the last author's clinical practice, where they were treated for behavioral problems. Two children with DLD were recruited via social media.

All children with DLD in the study were classified as having DLD by a multidisciplinary team at an audiological center in the Netherlands. To be diagnosed with DLD, adolescents had to meet specific criteria: (a) no significant hearing impairments, assessed by an audiologist; (b) nonverbal intelligence within the normal ranges, assessed by a psychologist; (c) no neurological issues; and (d) experiencing severe and persistent language difficulties significantly impacting communication effectiveness (SIAC, 2023). A speech-language therapist evaluated their language difficulties in multiple domains (speech, syntax, semantics, and pragmatics) based the Schlichting Test for Language Comprehension (Schlichting & Spelberg, 2010a), Schlichting Test for Language Production (Schlichting & Spelberg, 2010b), and Peabody Picture Vocabulary Test (Dunn et al., 2005; Dunn & Dunn, 1997). A child received a DLD diagnosis only in cases of a score of (a) ≥ 2 standard deviation below average in one of the four language domains, or (b) ≥ 1.5 standard deviation below average in two of the four language domains, or (c) ≥ 1 standard deviation below average in three of the four language domains. Deviations need to be present on at least two of the language tests. In children younger than four, diagnoses are established provisionally. Children with a provisional diagnosis must be monitored to ensure that diagnostics and treatment remain tailored to their needs. Children are classified definitively as having DLD, if language problems persist after intensive language therapy. All children included in the study received special education or in-patient treatment, suggesting that their language problems persist.

The TD children were recruited from mainstream schools in the Netherlands. They were matched individually to the children with DLD by a school for TD children based on age (less than 6 months difference), gender, and nonverbal intelligence. The latter criterion was also verified experimentally, allowing for maximally a 4-point difference in the raw score of Raven's standard progressive matrices (Raven, 2006; see Table 2). The TD children and children with DLD had no vision or hearing problems, had no classifications other than DLD, and were monolingual Dutch speakers. The names of all the participants were pseudonymized. The parents of all children signed an informed consent form.

**Table 2.** Descriptives at the First Test Session and Raw and Percentile (Perc.) Scores on Nonverbal Intelligence, Vocabulary and Syntax of the Children With Developmental Language Disorder (*n* = 5) and the Typically Developing Children (*n* = 5).

	Age (year; months)	Gender	Nonverbal intelligence		Vocabulary		Syntax	
			Raw	Perc.	Raw	Perc.	Raw	Perc.
Sophie	9;11	Girl	36	50–75	48	55	7	16
Sara	9;05		38	50–75	52	82.6	9	25
Milan	10;05	Boy	41	50–75	46	27.4	10	25
Mees	10;00		39	50–75	51	72.6	17	84
Anne	12;09	Girl	50	75–90	54	57.9	25	37
Amber	12;08		53	90–95	57	78.8	17	63
Lucas	10;05	Boy	41	50–75	45	27.4	4	5
Liam	10;02		44	50–75	54	84.1	13	37
Emma	11;06	Girl	31	10–25	51	50.0	8	5
Esther	11;01		33	10–25	52	57.9	10	9

*Note*. Nonverbal intelligence was assessed with the Standard Progressive Matrices (Raven, 2006). Vocabulary was assessed with the word meaning task from the second edition of the Revisie Amsterdamse Kinder Intelligentie test (RAKIT-2) (Resing et al., 2012); Syntax was assessed with the Sentence Assembly task from the fifth edition of the Clinical Evaluation of Language Fundamentals (CELF-5; Wiig et al., 2013, 2019).

### Tasks

**Word meaning structure**. Word meaning structure was measured using a newly developed Dutch version of the word meaning structure test (WMST; Toomela et al., 2020a), which was adapted for children with DLD. We employed a dynamic task of word meaning structure to distinguish three stages in the trajectory from everyday to logical concepts: (a) dominant logical concepts, (b) latent logical concepts, and (c) everyday concepts. The WMST comprises 18 items designed to assess word meaning structure using three types of open-ended questions. Six items required participants to provide definitions of specific concepts (e.g., “What is a school?”). Another set of six items asked participants to describe how the two concepts were similar (e.g., “Why do a cat and a dog go together?”). The last six items tasked children with selecting two out of three words that were related and explaining the basis for their selection. The words used in the WMST were drawn from the BasiLex corpus (Tellings et al., 2014), which lists the most commonly used words in texts designed for primary school children, separately per grade. We required a minimum of 30 occurrences in second-grade texts as the criterion for including the words in the task.

Initially, all 18 items were administered without assistance from the experimenter to elicit the participants’ dominant responses. Following the procedure outlined by Toomela et al. (2020a), a response was classified as a logical concept if it contained a hierarchically defined word or a relation between words (e.g., “a cat and a dog go together because they are animals”). If a child provided a logical concept in the initial response, the item was coded as a dominant logical concept. Subsequently, items that had not received a logical concept response in the first presentation (i.e., those generating everyday conceptual responses) were presented to the child again. Each child received two prompts crafted to elicit latent logical concepts (see Table 3). For each item, if the child did not offer a logical conceptual response after the first or second prompt, a potential solution was presented. At the end of the task, each item was coded as either a dominant logical concept (2 points), latent logical concept (1 point), or an everyday concept (0 points). The maximum dynamic scores were 36. Interrater reliability was calculated for the dominant responses, with a second rater, blinded to the study aims, and found to be almost perfect (Cohen's κ = .95). The Cronbach's alpha for the dynamic WMST in this study was .83.

**Table 3.** The Structure of Prompts Used in the Dynamic Word Meaning Structure Test.

	Word definitions	Similarities	Word pairs
First prompt	A *school* is a kind of … (e.g., a *place* where children can learn) [Een *school* is een … (e.g., een plek waar kinderen heen gaan om te leren)]	A *cat* and a *dog* are both … (e.g., animals) [Een *kat* en een *hond* zijn allebei … (e.g., dieren)]	You could also opt for a *bike* and a *car* as an example. Do you know why? A bike and a car are both … (e.g., means of transportation) [Je zou ook kunnen kiezen voor een *fiets* en een *auto*. Weet je waarom? Een fiets en een auto zijn allebei … (e.g., vervoersmiddelen)]
Second prompt	What other things are like *a school*, and could you tell me why in only a single word? [Wat voor andere dingen zijn zoals een *school*, en kan je in één woord zeggen waarom?]	Could you tell me how a *cat* and a *dog* are alike in only a single word? [Zou je me in één woord kunnen zeggen waarom een *kat* en een *hond* bij elkaar horen?]	Could you tell me in only a single word how the *bike* and the *car* are alike? [Zou je me in één woord kunnen zeggen waarom de *fiets* en de *auto* bij elkaar horen?]

**EF.** EF was measured using the Zoo Map Task (Emslie et al., 2003). We opted for this task because it is an ecologically valid test of planning ability as a higher order EF. This includes a control subtask that draws on planning to a more limited degree. Participants were tasked with drawing a route on a zoo map, with the objective of visiting specific animals while adhering to designated rules (e.g., not retracing a white path). This task encompassed two subtasks: one in which the animals were listed but without instructions for the correct visiting order, and another in which the children were given the specific visiting order. Performance on both subtasks was assessed by tallying the number of sites visited in the correct sequence with deductions for rule-related errors. To assess everyday difficulties related to general EF, we included the Dutch version of the Behavior Rating Inventory for Executive Function (BRIEF; Gioia et al., 2000; Huizinga & Smidts, 2009) using only the total score. This is a parent-rated questionnaire consisting of 75 items rated on a 3-point Likert-scale (1 = “*never*,” 2 = “*sometimes,*” and 3 = “*often*”). We report only the total score (maximum possible score = 225).

**ToM.** ToM was assessed using the ToM test (Steerneman et al., 2003), a reliable (Cronbach's alpha is 0.88 in TD children; Steerneman et al., 2003) Dutch test suitable for children with DLD. It covers many aspects of ToM development, including elementary forms of ToM, such as emotion recognition, and manifestations of first- and second-order ToM. In the ToM test, children responded to 36 questions about their mental states based on 14 vignettes accompanied by images on A3-sized paper sheets. Participants could attain a maximum score of 36.

The Frith-Happé Animations (Abell et al., 2000) were also included because they provide more insight into spontaneous ToM processing in children. The participants narrated video clips featuring moving triangles. The clips included four animations involving large red and small blue triangles, with one character reacting to the mental states of the other (e.g., persuading one another). Each clip lasted between 35 and 45 s. Participants were instructed to describe the events in the clip immediately after viewing, and their narrations were scored (0, 1, or 2) in two dimensions. The accuracy was based on the degree to which the descriptions met the intended meaning of the clip. In addition, a mental state term score was computed based on whether the description included a lower (1 point) or higher (2 points) level mental state word. The maximum scores, ranging from 0 to 8, were computed by summing the scores of the four clips.

Finally, the parents of the participating children were given the Bermond-Vorst Alexithymia Questionnaire (BVAQ; Bermond & Vorst, 1996) to determine their children's ToM. The parents rated their children on 40 items graded on a 5-point Likert scale (1 = “*definitively applies*,” 5 = “*in no way applies*”) that assessed their children's ability to identify, reflect upon, and describe their emotions and fantasies. The reliability of the BVAQ is reported to be .85 (Vorst & Bermond, 2001).

**Behavioral problems**. Behavioral problems were assessed using the Dutch version of the Child Behavior Checklist (CBCL) for children aged 6–18 (Achenbach, 1991; Verhulst & van den Ende, 2013). This is a reliable (Cronbach's alpha for the questionnaire has been shown to be .99 for TD boys and girls; Verhulst & van den Ende, 2013) parental questionnaire that distinguishes between internalizing and externalizing behavioral problems. The questionnaire comprises 113 items regarding a child's behavior (e.g., “argues a lot”), rated based on frequency. The items were rated on a 3-point Likert-scale (0 = “*not true*,” 1 = “*a bit true or sometimes true*,” and 2 = “*very true or often true*”). The full questionnaire can be divided into eight subscales, namely (a) anxious/depressed, (b) withdrawn/depressed, (c) somatic problems, (d) Social Problems, (e) thought problems, (f) attention problems, (g) rule-breaking behavior, (h) aggressive behavior, and (i) other problems. Subscales 1–3, and 7–8 can be combined into two composite scales, namely internalization and externalization problems respectively. The maximum possible CBCL score is 226.

### Procedure

Children with DLD participated in three 30-min sessions conducted either in their homes or in the clinic where they were recruited. The TD children were tested by the first author in a quiet room at their school. All tests were administered by the first author and the tasks were administered in a fixed order. The first session consisted of the Zoo Map Task, Standard Progressive Matrices, and Sentence Assembly task from the Clinical Evaluation of Language Fundamentals (fifth edition; CELF-5; Wiig et al., 2013, 2019). The second session consisted of a word meaning task from the second edition of the Revisie Amsterdamse Kinder Intelligentie test (RAKIT-2; Resing et al., 2012), followed by the Frith-Happé Animations and ToM Test. The third session involved the dynamic WMST. Additionally, anamnestic interviews with the parents were conducted to gather recollections of case history information encompassing the participants’ socioeconomic status, language-related issues within the family, year of DLD classification, social-emotional functioning, behavioral problems, and the nature of the child's language difficulties. Audio recordings were made during the anamnestic interviews for later transcription, and the experimenter documented the observations throughout the testing sessions.

## Results

### Scores of children with DLD and TD children on EF, ToM, and behavioral problems

Table 4 presents the individual scores for the children with DLD and TD children on the neuropsychological tasks and Table 5 displays the individual scores on the questionnaires. Due to the small sample size, it was not feasible to conduct statistical analyses to determine the differences between the groups. Nevertheless, we computed Cohen's *d* to tentatively interpret the magnitude of the differences between the groups (Cohen, 1988). There was a large difference between the children on the Zoo Map Task (*d *= −1.17). Children with DLD scored moderately lower than TD children on the ToM Test (*d *= −.66). Contrastingly, children with DLD scored slightly lower than TD children in the number of mental state terms (*d *= −.31), and moderately higher on accuracy (*d *= .76) in the Frith-Happé Animations. Furthermore, parents of children with DLD reported more differences in everyday EF difficulties on the BRIEF (*d *= 1.19) and negligible differences in the ability to identify and analyze emotions on the BVAQ (*d *= −.10). Regarding behavioral problems, there were moderate differences in internalizing problems (*d *= .54), and negligible differences in externalizing problems (*d *= .12).

**Table 4.** Individual Raw Scores, Individual Percentile Scores, and Mean Group Scores of the Children With Developmental Language Disorder (*n* = 5) and the Typically Developing Children (*n* = 5) on the Neuropsychological Tests.

	Pair 1		Pair 2		Pair 3		Pair 4		Pair 5		Groups			
	DLD	TD	DLD	TD	DLD	TD	DLD	TD	DLD	TD	DLD		TD	
	Sophie	Sara	Milan	Mees	Anne	Amber	Lucas	Liam	Emma	Esther	*M*	*SD*	*M*	SD
EF (ZMT1)	0 (20.2–30.9)	4 (69.2–79.8)	1 (12.2–20.2)	7 (79.8–87.7)	8 (>87.8)	3 (30.9–43.4)	−3 (3.3–6.7)	6 (69.2–79.8)	3 (43.4–56.6)	8 (>93.3)	1.80	4.09	5.60	2.07
EF (ZMT2)	8 (>56.6)	8 (>56.6)	3 (0.2–0.6)	8 (>69.2)	8 (>56.6)	8 (>56.6)	8 (>69.2)	8 (>69.2)	7 (>69.2)	8 (>69.2)	6.80	2.17	8.00	0.00
ToM (ToM test)	32 (15.9–50)	29 (6.7–15.9)	31 (15.9–50)	36 (>98)	33 (15.9–50)	35 (50–84.1)	30 (6.7–15.9)	32 (15.9–50)	31 (6.7–15.9)	32 (15.9–50)	31.40	1.14	32.80	2.77
ToM (FHA accuracy)	4	1	7	3	6	3	0	4	3	3	40	2.74	2.80	2.10
ToM (FHA MSTs)	1	4	3	2	4	3	4	2	1	4	2.60	1.52	3.00	1.00

*Note*. ZMT = Zoo Map Task; FHA = Frith-Happé Animations; MSTs = mental state terms; EF = Executive Functioning; ToM = Theory of Mind.

When available, the percentile scores of the children with DLD are presented between parentheses.

**Table 5.** Individual Raw Scores, Individual Percentile Scores, and Mean Group Scores of the Children With Developmental Language Disorder (*n* = 5) and the Typically Developing Children (*n* = 5) on the Parental Questionnaires.

	Pair 1		Pair 2		Pair 3		Pair 4		Pair 5		Groups			
	DLD	TD	DLD	TD	DLD	TD	DLD	TD	DLD	TD	DLD		TD	
	Sophie	Sara	Milan	Mees	Anne	Amber	Lucas	Liam	Emma	Esther	*M*	*SD*	*M*	*SD*
Executive functioning (BRIEF)	125 (54)	99 (16)	149 (73)	92 (5)	136 (73)	97 (24)	141 (62)	144 (66)	138 (82)	142 (82)	137.80	8.70	114.80	25.88
Theory of Mind (BVAQ)	128	136	91	141	122	83	125	122	127	101	118.60	15.60	116.60	24.36
Internalizing problems (CBCL)	3 (<50)	5 (50–69)	5 (50–69)	1 (<50)	26 (>98)	3 (<50)	3 (<50)	9 (84–93)	23 (>98)	17 (93–98)	12.00	11.49	7.00	6.32
Externalizing problems (CBCL)	1 (<50)	6 (50–69)	4 (50–69)	4 (50–69)	9 (69–84)	1 (<50)	2 (<50)	7 (69–84)	16 (84–93)	10 (69–84)	6.20	6.42	5.60	3.36

*Note*. BRIEF = Behavior Rating Inventory for Executive Function; CBCL = Child Behavior Checklist; BVAQ = Bermond-Vorst Alexithymia Questionnaire.

The percentile scores are presented between parentheses.

### Word meaning structure in children with DLD and TD children

**Figure 1.**
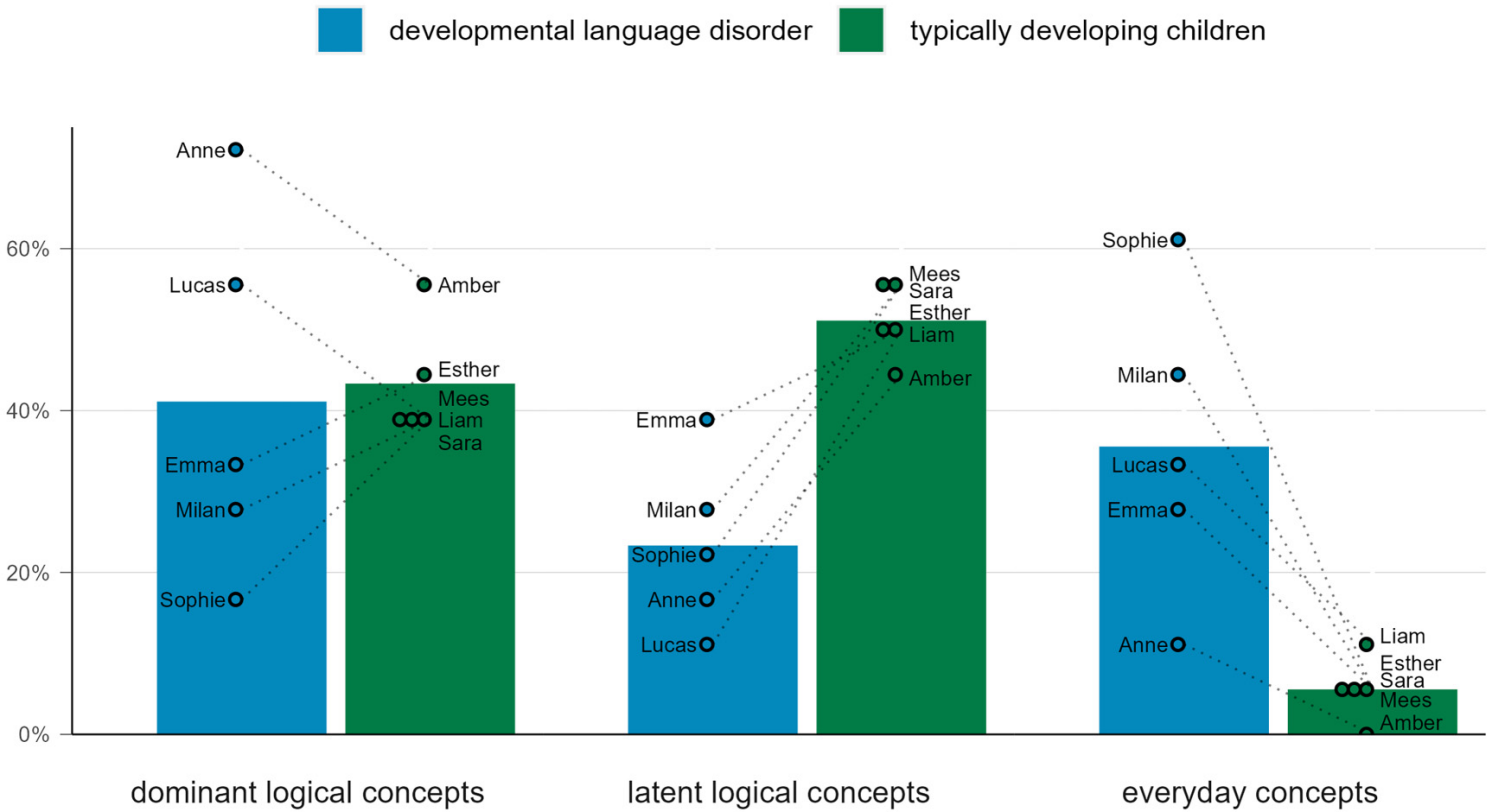
The distribution of response categories on the dynamic word meaning structure test by the children with developmental language disorder (*n* = 5) and the typically developing children (*n* = 5).

The first research question was whether there was a difference in word meaning structure between children with and without DLD. Figure 1 illustrates the group means and individual scores of the children across the three dynamic WMST categories. On average, children with DLD and TD children produced roughly the same (*d *= −.13) percentage of dominant logical concepts (DLD: *M *= 41.1%, *SD *= 22.4%, TD: *M *= 43.3%, *SD *= 7.3%), but children with DLD produced fewer (*d *= −3.37) latent logical concepts (DLD: *M *= 23.3%, *SD *= 10.7%, TD: *M *= 51.1%, *SD *= 4.7%), and more (*d *= 2.22) everyday concepts (DLD: *M *= 35.6%, *SD *= 18.7%, TD: *M *= 5.6%, *SD *= 3.9%) after the two prompts. Based on the total dynamic scores, there was a large (*d *= 1.10) difference between children with DLD (*M *= 19, *SD *= 7.18) and TD children (*M *= 24.8, *SD *= 1.92). These findings suggest that children with DLD have poorer word meaning development than TD children. Moreover, a wider dispersion of word meaning structure scores was observed in the DLD group than the TD group.

### Associations among word meaning structure, EFs and ToM in children with DLD and TD children

**Figure 2.**
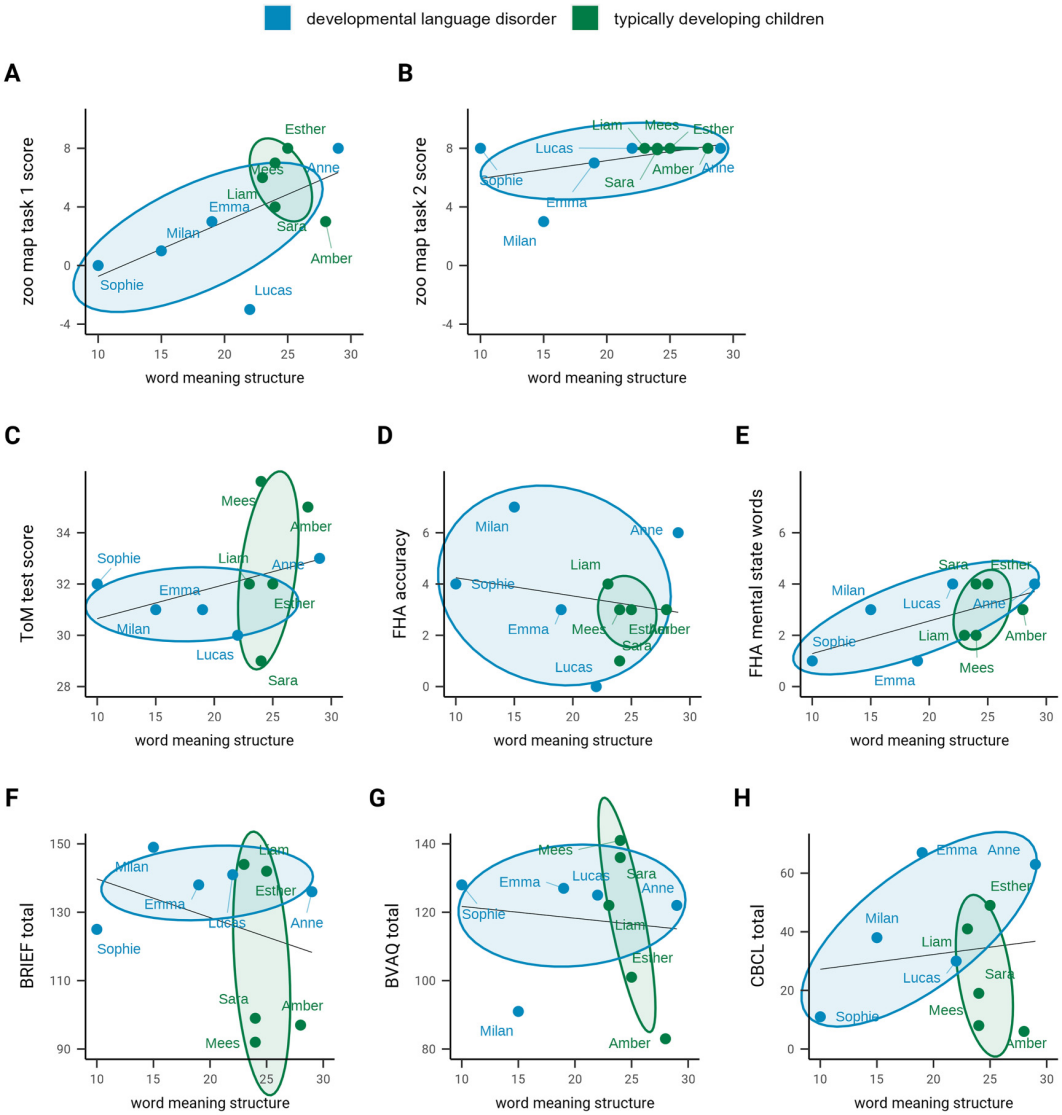
Scatterplots showing the relationships between word meaning structure on the one hand, and behavioral tests and questionnaires for executive functioning, theory of mind, and internalizing and externalizing behavioral problems on the other in the children with developmental language disorder (*n* = 5) and the typically developing children (*n* = 5).

Note. EF = Executive Functioning; ZMT = Zoo Map Task; ToM = Theory of Mind; FHA = Frith-Happé Animations; BRIEF = Behavior Rating Inventory for Executive Function; CBCL = Child Behavior Checklist; BVAQ = Bermond-Vorst Alexithymia Questionnaire.

The second research question was how word meaning structure in children with and without DLD is related to EF, ToM, and behavioral problems. Figure 2 presents scatterplots showing the individual scores of the children, as well as the relationships between word meaning structure, and EF, ToM, and behavioral problems. Judging by the scatterplots, among the sample as a whole, higher WMST scores are strongly associated with higher scores on Zoo Map Task 1 (*r *= .59; see Figure 2A). On Zoo Map Task 2, ceiling effects render the association uninformative (Figure 2B). Thus, word meaning structure appears to be positively related to EF. Moreover, dynamic WMST scores were positively related to performance on the ToM test (*r *= .33; see Figure 2C) and the number of mental state words mentioned after watching the clips from the Frith-Happé Animations (*r *= .60; see Figure 2E) but negatively related to the Frith-Happé Animations accuracy scores (*r *= −.20; see Figure 2D).

In addition, dynamic WMST scores were negatively related to scores on the BRIEF (*r *= −.30; see Figure 2F) and BVAQ (*r *= −.10, see Figure 2G). This implies that a higher level of word meaning structure is usually paired with lower levels of everyday EF problems and difficulties in identifying, analyzing, and describing emotions and fantasies. Thus, on balance, word meaning structure appears to be positively related to EF and ToM. However, there was a positive relationship between the dynamic WMST scores and the internalizing (*r *= .29; see Figure 2H) and externalizing scores (*r *= .22; see Figure 2I) of the CBCL scores, which indicates that a more developed word meaning structure is generally paired with more behavioral problems.

### Case studies

Below, we present case studies of individual children with DLD, in contrast to TD children and norm scores. For each case, we first describe the general case history based on anamnestic interviews. Thereafter, we describe the performance of the children on neuropsychological tasks, remarking on cases in which individual children differ from the general group patterns.

**Sophie (girl, aged 9;11, DLD), matched with Sara (girl, aged 9;05, TD).** From an early age, Sophie's parents noticed that she had attention problems and could not understand verbal instructions. Clinical tasks revealed no intelligence or hearing irregularities. Her problems were primarily verbal, and she was eventually classified as having DLD. At school, Sophie's performance has been stagnant for some years. Although she has been able to learn facts through repetition, abstract subjects such as mathematical stories and reading comprehension have been difficult. Sophie has become increasingly aware of her shortcomings, sometimes to her own frustration. Nevertheless, Sophie is described by her parents as a joyful child who is liked by her peers.

Although her vocabulary and syntax scores were relatively normal, Sophie attained the lowest dynamic WMST score in the sample. She produced 16.7% dominant logical concepts (Sara: 38.4%), 22.2% latent logical concepts (Sara: 55.6%), and 61.1% everyday concepts (Sara: 5.6%; see Figure 1). As expected, based on these low WMST scores, she also had difficulties with EF, as indicated by her low score on Zoo Map Task 1 (see Figure 2). In Zoo Map Task 1, Sophie took some time to plan but then appeared to give up and went directly toward the animal nearest to the exit, forcing her to break the task rules. In contrast, Sara took a long time to preplan and attained a higher score on Zoo Map Task 1. Sophie's ToM appeared to be relatively normal for her age; indeed, she scored higher than Sara on the ToM test and offered more accurate descriptions in Frith-Happé Animations. Finally, Sophie's CBCL scores did not reveal internalizing or externalizing behavioral problems.

**Milan (boy, aged 10;05, DLD), matched with Mees (boy, aged 10;00, TD).** Milan was described by his parents as a sweet and helpful boy. In his early years, he had several medical problems, including kidney dysfunction and otitis media. The late emergence of his first words at the age of three raised concerns that resulted in a DLD classification that same year. Since then, his parents and speech therapists have observed a range of language problems, including speech comprehension, reading, writing, word finding, and, most recently, understanding jokes and figurative language. After being classified as having DLD, Milan attended regular education with outpatient services. At school, Milan increasingly fell behind his classmates. He had difficulty grasping abstract matters such as clock reading, arithmetic, and understanding historical events. At times, Milan was bullied and excluded from activities by his peers, and over the years, his social circle has decreased to a single friend. His behavioral problems were compounded by an increasing awareness of his cognitive shortcomings compared with his peers.

Of Milan's answers on the dynamic WMST (see Figure 1), 27.8% were dominant logical concepts (Mees: 38.9%), 27.8% were latent logical concepts (Mees: 55.6%), and 44.4% were everyday concepts (Mees: 5.6%). He attained low scores on both versions of the Zoo Map Task and scored higher than normal on the behavioral regulation and metacognition scales of the BRIEF. Contrastingly, his ToM abilities, appeared normal in the tasks. Nevertheless, Milan's parents indicated that there were ToM difficulties, specifically in understanding the meaning of nonliterate language, such as figurative speech and jokes. Overall, Milan appeared to have some EF difficulties, whereas ToM seemed to be relatively intact. He scored within the normal range for both internalizing and externalizing behavioral problems, on the CBCL. However, he scored in the subclinical range on the social and thought problems scales. In contrast, Mees attained normal to high scores on all questionnaires and tasks for EF and ToM, apart from the number of mental state words mentioned in the Frith-Happé Animations.

**Anne (girl, aged 12;09, DLD), matched with Amber (girl, aged 12;08, TD).** Anne was described by her mother as an amiable girl who is passionate about her hobbies. She was delivered through a cesarean section due to a serious heart abnormality, for which she underwent corrective surgery at the age of one. In recent years, Anne's health has improved, although stressful times still regularly resulted in low energy levels. By the age of two and a half, Anne was classified as having DLD because she had not yet started speaking. When her speech emerged shortly afterward, others struggled to comprehend her. Anne's school achievements have fluctuated, but her grades, including those related to language development, have recently shown considerable improvement, and she secured a significant role in her school musical. However, Anne continues to have difficulty maintaining friendships, and she is regularly excluded by her peers from social situations.

Anne attained the highest dynamic WMST score in the sample (see Figure 1). She mentioned 72.2% dominant logical concepts (Amber: 55.6%), 16.7% latent logical concepts (Amber: 44.4%), and 11.1% everyday concepts (Amber: 0%). For both Anne and Amber, high WMST scores were paired with relatively high scores on the cognitive tasks (see Figure 2). On the Zoo Map Task, Anne did not take much time planning but attained the maximum score on both versions. Anne and Amber attained average ToM test scores for their ages, although Amber scored slightly higher. Anne's scores on the tasks and questionnaires for word meaning structure, EF, and ToM were at odds with her difficulties in behavioral problems revealed in the anamnestic interviews and her CBCL scores. She scored in the clinical range for internalizing problems and in the normal range for externalizing problems.

**Lucas (boy, aged 10;5, DLD) matched with Liam (boy, 10;02, TD).** Lucas was described by his parents as a happy and active boy with great curiosity. His early years were marked by recurrent otitis media until the age of five, sometimes accompanied by hearing difficulties. Lucas began speaking at the age of three, and his parents soon noticed that he frequently struggled to comprehend verbal instructions. Around the age of four and a half, Lucas was classified as having DLD. His family has a history of dyslexia, and his parents suspect that Lucas may be dyslexic, given his reading and writing challenges. Although Lucas's vocabulary has expanded considerably, syntax has remained a challenge for him. In social situations, Lucas has always faced difficulties in understanding others. His mother worries that Lucas could be manipulated because of his efforts to be liked by his peers.

Lucas scored relatively high on the WMST (see Figure 1), with 55.6% dominant logical concepts (Liam: 38.9%), 11.1% latent logical concepts (Liam: 50.0%), and 33.3% everyday concepts (Liam: 11.1%). This outcome contrasts with the Zoo Map Task and ToM Test scores (see Figure 2). Before drawing routes on the zoo maps, Lucas spent less time planning than Liam. In contrast to most of the other children in the present study, Lucas marked the animals he had visited on the instruction paper. On the BRIEF, Lucas scored in the clinical range on the attention scale, corroborating his parents’ remarks that he was easily distracted and often did not check his work for errors. On the Frith-Happé Animations, he used a mental word in each video, but simultaneously gave inaccurate descriptions for each video. For example, Lucas described a video depicting “mocking” as follows: “They were lost, and that's … she found that … and then she went all out looking. And then suddenly, she had found it.” His parents did not report internalizing or externalizing behavioral problems on the CBCL, although Lucas scored in the subclinical range on the Social Problems scale.

**Emma (girl, aged 11;6, DLD), matched with Esther (girl, 11;01, TD).** Emma was described by her parents as a shy, but caring and inventive girl. She was born after an unproblematic pregnancy. An early diagnostic program in Emma's daycare center revealed poor speech and communication skills. She was classified as having DLD at the age of three. She likes playing sports and tinkering, and she can become immersed in her activities, sometimes for weeks on end. In social situations, Emma is regularly excluded, and has difficulty maintaining friendships with peers. She also experiences mood swings.

Emma mentioned 33.3% dominant logical concepts (Esther: 44.4%), 38.9% latent logical concepts (Esther: 50.0%), and 27.8% everyday concepts (Esther: 5.6%) in the WMST (see Figure 1). Her scores on both versions of the Zoo Map Task were normal, although lower than those of Esther. However, Emma scored lower on everyday manifestations of EF, as signified by her BRIEF scores, especially in the behavioral regulation domain. According to her mother, Emma's difficulties have been most pronounced in adapting to new situations, especially when transitioning to unstructured situations, such as school holidays. In addition to these everyday EF difficulties, Emma's ToM skills appear to be underdeveloped. She scored below average on the ToM test and mentioned only a single mental state word when describing the Frith-Happé Animations. However, Emma's parents remarked that Emma is capable of reasoning about the mental states of others when prompted to do so. On the CBCL, Anne's scores fell within the clinical range for internalization problems, and her externalization score was also high, albeit within the normal range.

## Discussion

The primary aim of the current study was to take the first step in understanding word meaning structure in children with DLD and how this relates to EF, ToM, and behavioral problems. Word meaning structure was assessed without prompts (statically) and with prompts from the experimenter (dynamically) to measure dominant and latent logical concepts, respectively. These results tentatively suggest that there are differences in word meaning structure between children with DLD and TD children. In addition, word meaning structure scores appeared to relate positively to EF and ToM. These findings were analyzed within the context of the children's individual case histories.

### Word meaning structure in children with DLD

The first research question answered in this study was whether there was a difference in word meaning structure between children with and without DLD. We used a dynamic assessment approach to distinguish among (a) dominant logical concepts, (b) latent logical concepts, and (c) everyday concepts. Based on the small differences in logical concepts produced without the experimenter's assistance, we concluded that the pairs of children differed little in terms of dominant logical concepts. However, with the introduction of prompts, the distinctions between the groups became more noticeable. Children with DLD responded more frequently with everyday concepts after the second prompt, suggesting that they had developed fewer latent logical concepts than TD children. This fits with studies showing that dynamic assessment is more predictive of language impairment in other language domains such as word learning (Camilleri & Law, 2014; Kapantzoglou et al., 2012) and syntactic structure (Hasson et al., 2012). In summary, even though logical concepts may be equally dominant in the present task setting, children with DLD may be delayed in their word meaning structure development.

Although the present study was the first to assess word meaning structure in children with DLD, the results align with those of previous studies on metalinguistic abilities in children with DLD. Studies have shown that children with DLD tend to provide more concrete definitions (but not necessarily in the form of definitions; Dosi & Gavriilidou, 2020, 2021) and have more difficulty comprehending categorical relationships between words than TD children (e.g., Acosta Rodríguez et al., 2020). These findings extend earlier work showing differences in the breadth of word knowledge (e.g., McGregor et al., 2013), showing that children with DLD do not only differ from TD children in the amount of content associated with a word, but that there are also differences in the relationships between words and their meaning, that is, the representations they refer to.

### Word meaning structure in relation to EF, ToM, and behavioral problems

The second research question was whether word meaning structure relates to EF, ToM, and behavioral problems. The results indicated group-level relationships between word meaning structure, EF, and ToM. The graphs do not show a negative relationship between word meaning structure and behavioral problems in the present sample. If there is a relationship between word meaning structure and behavioral problems, this has been overshadowed by other factors in this study. Nevertheless, the associations between word meaning structure, EF, and ToM align with the theory of semiotic mediation, positing that language plays a role in organizing sensory experiences (Toomela, 2020a). Word meaning structure development, as it progresses from everyday concepts to logical concepts, and thereby gradually detaches from sensory experiences, enhances the possibility of employing language to organize sensory experiences. Theoretically, this should benefit EF and ToM (see Camminga et al., 2021). Although research on these relationships remains scarce, some studies have shown that reliance on logical concepts is related to EF in TD children (Dosi, 2021) and adults (Toomela et al., 2020b). Nevertheless, despite the general relationships found in this study, individual-level disparities warrant further discussion. Importantly, many factors, most of which lie outside the scope of the case studies, can account for these disparities. Thus, these explanations are highly tentative and primarily intended to propose new testable hypotheses.

Lucas produced many dominant logical concepts but scored low on EF and ToM. There are several possible reasons for this discrepancy. First, there may be no necessary relationships among word meaning structure, EF, and ToM. Alternatively, we speculate that Lucas may have learned to respond with logical concepts to questions similar to those of the WMST (e.g., in an educational context; Kaminske et al., 2020). This phenomenon may be more common in children with DLD. Unlike age-matched TD children, logical concepts are not related to EF in children with DLD, suggesting that they may not rely on logical concepts in noneducational contexts to the same extent (Dosi, 2021). Simultaneously, this suggests that the extent of prior exposure to logical concepts in education or in interventions targeting areas related to word meaning structure (e.g., on lexical-semantic representations) accounts for some differences in word meaning structure scores but may not necessarily imply reliance on logical concepts in particular EF and ToM tests. However, Sophie scored low on word meaning structure but normal on ToM. Given that she engages in many social situations, this might indicate that her verbal concepts in the social and emotional domains are well-developed compared with other domains of knowledge. This is certainly possible given the domain-specific nature of word meaning structure development (Toomela, 2020b; Vygotsky, 1934).

### Limitations and strengths

Compared with larger studies, the present study was limited by the fact we could not conduct statistical tests that would allow to control for confounding factors and to generalize the results to a larger population of children with DLD. Moreover, children with DLD are a heterogeneous group, and difficulties in word meaning structure, EF, and ToM possibly apply only to a subtype of children with DLD (e.g., expressive vs. expressive-receptive subtypes of DLD)—even at the group level no differences were found in certain ToM measures or in internalizing behavioral problems. An additional limitation is that we did not assess prior participation in interventions in areas such as lexical-semantic representations, which could feed into word meaning structure. Finally, the present study focused exclusively on the weaknesses in children with DLD. From a neurodiversity perspective (Hobson et al., 2024), it should be noted that children with DLD may have relative strengths such as, coping skills (McGregor et al., 2023) and pro-social behavior (Dubois et al., 2020).

A strength of the current study is that the dynamic assessment approach allowed us to make a more fine-grained distinction in word meaning structure than a static test would have allowed. Another strength is that the individual-level analysis allowed us to conjecture why individual children deviated from the average. Compared to single-case studies, the multiple case design children better highlighted the heterogeneity in the DLD population (e.g., van Weerdenburg et al., 2006).

### Implications

This study highlights potential new questions for future research. Specifically, by suggesting differences in word meaning structure and showing how these relate to EF and ToM, this study serves as a basis for future research. It suggests that future studies should focus on the relationships between EF and ToM abilities and word meaning structure in children with DLD. While the present study was exploratory, future studies should focus on specific aspects of EF and ToM as both are multidimensional constructs (Diamond, 2013; Westby & Robinson, 2014). The results of the present study may inform the development of new interventions or existing interventions that target areas that feed into word meaning structure development (e.g., lexical representations), and show how such interventions may enhance EF and ToM in addition to communicative abilities. Word meaning structure emphasizes qualitative differences in the representation of word meanings and suggests that promoting the acquisition of later levels of word meaning structure may support more effective EF and ToM. For example, in the domain of emotion understanding, therapists could help children establish the general concept of “emotion” to help children more clearly different emotions. As EF is not tied to any specific conceptual domain, the acquisition of domain-general logical concepts in education may be more relevant.

## Conclusions

The present study is the first to examine word meaning structure in children with DLD. Although our study should be seen as a first step, we tentatively conclude from our results that there is a difference in word meaning structure development between children with DLD and those with TD. Based on a static approach (i.e., without experimenter assistance), there was scant difference in word meaning structure. However, based on a dynamic approach (i.e., with experimenter assistance), clearer differences in word meaning structure emerged between children with and without DLD. Moreover, word meaning structure seemed to be related to aspects of EF and ToM. Overall, the present study tentatively suggests that some of the EF and ToM difficulties and, by extension, the behavioral problems in children with DLD may be related to their word meaning structure. Our multiple case design allowed us to analyze deviations at the individual level and explore potential explanations. New research questions can emerge from these that can be answered in future studies. Specifically, future studies should address the relationship between word meaning structure and behavioral problems and whether EF and ToM are better predicted by domain-specific (e.g., using only emotion words) rather than domain-general word meaning structure tasks. This research path has the potential to shed light on the complex interplay between language development, EF, and ToM in children with and without DLD.

## Notes

1. Note that this differs from the notion of a lexical-semantic network. Such theories consider development in terms of the number of connections between words (children with DLD present differences in that regard too; for example, Sheng & McGregor, 2010), while the concept of word meaning structure considers qualitative development in terms of the types of relationships among words and between words and referents, and assumes that words actively organize conceptual knowledge.
